# From Behaviour to Biology: An Educational Framework for Distinguishing bvFTD From Primary Psychiatric Disorders

**DOI:** 10.1192/bjo.2026.11151

**Published:** 2026-06-30

**Authors:** Similoluwa Adewale

**Affiliations:** Imperial College London, London, United Kingdom

## Abstract

**Aims:**

Behavioural-variant frontotemporal dementia (bvFTD) is a neurodegenerative condition that typically presents in mid-life with early behavioural and personality change,often in the absence of marked global cognitive impairment. This clinical profile substantially overlaps with primary psychiatric disorders (PPDs), contributing to frequent misdiagnosis and diagnostic delay. Cognitive screening is routinely used in early assessment, yet the choice and interpretation of tools is often clinician-dependent and inconsistently taught. This work aims to explore how limitations in commonly used cognitive screening tools contribute to diagnostic uncertainty between bvFTD and PPDs, and to emphasise the importance of structured education on when and how to escalate assessment to appropriate neuroimaging.

**Methods:**

A case-based educational analysis was undertaken using a representative behavioural presentation as a teaching framework. The current literature on cognitive screening tools, including the Mini-Mental State Examination, Addenbrooke’s Cognitive Examination, and Montreal Cognitive Assessment, was reviewed with attention to their sensitivity for detecting executive and social cognitive dysfunction. Evidence relating to neuroimaging modalities used in suspected bvFTD was also examined, including structural MRI, FDG-PET, and amyloid PET, with emphasis on their relative diagnostic roles in resolving behavioural diagnostic ambiguity.

**Results:**

Global cognitive screening tools, which are weighted towards memory and orientation, demon3strate limited sensitivity for early bvFTD, where executive and social cognitive deficits often predominate. This contributes to frequent misclassification as psychiatric illness when screening results are interpreted in isolation. Structural MRI provides supportive but often non-discriminatory findings in early disease, particularly where subcortical involvement may precede overt cortical atrophy. FDG-PET offers greater diagnostic utility by identifying frontal and anterior temporal hypometabolism; however, findings are not entirely specific and require careful clinical correlation due to the incidenceof false positives. Amyloid PET does not directly differentiate bvFTD from PPDs but serves an important exclusionary role by ruling out Alzheimer’s disease in atypical behavioural presentations.

**Conclusion:**

Effective differentiation of bvFTD from primary psychiatric disorders requires more than symptom recognition alone. Improved education in cognitive screening–specifically tool selection, interpretation, and limitations–combined with clearer escalation strategies for neuroimaging, is essential. Future educational and diagnostic approaches may benefit from integrating quantitative imaging techniques with emerging biological markers, including genetic testing in early-onset presentations and fluid biomarkers such as neurofilament light chain. Embedding these principles within medical training supports a multimodal diagnostic framework, with important implications for reducing misdiagnosis and improving patient outcomes.

